# TRIB3 regulates FSHR expression in human granulosa cells under high levels of free fatty acids

**DOI:** 10.1186/s12958-021-00823-z

**Published:** 2021-09-09

**Authors:** Nan Wang, Chenchen Si, Lan Xia, Xian Wu, Sheng Zhao, Huihui Xu, Zhide Ding, Zhihong Niu

**Affiliations:** 1 Department of Gynecology and Obstetrics, Ruijin Hospital Affiliated with the Medical School of Shanghai Jiao Tong University, Shanghai, 200025 China; 2 Department of Histology, Embryology, Genetics and Developmental Biology, Shanghai Key Laboratory for Reproductive Medicine, Shanghai Jiao Tong University School of Medicine, Shanghai, 200025 China

**Keywords:** Tribbles pseudokinase 3 (TRIB3), Follicle stimulating hormone receptor (FSHR), Akt/GSK3β pathway, Palmitic acid, Human granulosa cells

## Abstract

**Background:**

Granulosa cells (GCs) in cumulus oophorus highly express follicle stimulating hormone receptor (FSHR), which is the most important mediator of both estradiol synthesis and oocyte maturation. Obese women have elevated free fatty acids (FFAs) levels in their follicular fluids and decreased FSHR expression in GCs, which is related to an altered protein kinase B/glycogen synthase kinase 3β (Akt/GSK3β) signaling pathway*.* Such FFA increases accompany 3-fold rises in pseudokinase 3 (TRIB3) expression and reduce the Akt phosphorylation status in both the human liver and in insulinoma cell lines. Therefore, in a high FFA environment, we determined if TRIB3 mediates regulation of FSHR via the Akt/GSK3β signaling pathway in human GCs.

**Methods:**

GCs from women undergoing in vitro fertilization were collected and designated as high and low FFAs cohorts based on their follicular fluid FFA content. GCs with low FFA levels and a human granulosa-like tumor (KGN) cell line were exposed to palmitic acid (PA), which is a dominate FFA follicular fluid constituent. The effects were assessed of this substitution on the Akt/GSK3β signaling pathway activity as well as the expressions of TRIB3 and FSHR at both the gene and protein levels by qPCR, Western blot and immunofluorescence staining analyses. Meanwhile, the individual effects of *TRIB3* knockdown in KGN cells and p-AKT inhibitors were compared to determine the mechanisms of FFA-induced FSHR downregulation.

**Results:**

The average FSH dose consuming per oocyte (FSH dose/oocyte) was elevated and Top embryo quality ratio was decreased in women with high levels of FFAs in their follicular fluid. In these women, the GC TRIB3 and ATF4 protein expression levels were upregulated which was accompanied by FSHR downregulation. Such upregulation was confirmed based on corresponding increases in their gene expression levels. On the other hand, the levels of p-Akt decreased while p-GSK3β increased in the GCs. Moreover, *TRIB3* knockdown reversed declines in FSHR expression and estradiol (E2) production in KGN cells treated with PA, which also resulted in increased p-Akt levels and declines in the p-GSK3β level. In contrast, treatment of *TRIB3*-knockdown cells with an inhibitor of p-Akt (Ser473) resulted in rises in the levels of both p-GSK3β as well as FSHR expression whereas E2 synthesis fell.

**Conclusions:**

During exposure to a high FFA content, TRIB3 can reduce FSHR expression through stimulation of the Akt/GSK3β pathway in human GCs. This response may contribute to inducing oocyte maturation.

**Supplementary Information:**

The online version contains supplementary material available at 10.1186/s12958-021-00823-z.

## Background

Cumulus oophorus bear mature follicles that are composed of a secondary oocyte and surrounded by granulosa cells (GCs, derived from follicular cells). Their formation is one of the necessary conditions for natural fertilization to occur. Normal follicular growth depends on both timely endocrine stimulation and their nutrient and energy statuses [1]. GCs can foster oocyte support localized in the center of the follicle by providing the nutrients via the zona pellucida until oocyte maturation or ovulation. In clinics, folliculogenesis is severely impaired in some patients afflicted with a metabolic disease such as obesity [2]. As a characteristic marker of nutritional imbalances, both nutritional deprivation and excess were found to have the detrimental effects on non-esterified fatty acids (NEFAs) or free fatty acids (FFAs) and in turn on folliculogenesis in both human and animal models [3]. During in vitro fertilization (IVF), obese women have high FFAs in their follicular fluid [4] and lower estradiol (E2) levels in their serum [5], and usually require more gonadotrophin to induce ovarian stimulation [6–8]. On the other hand, both in vitro and in vivo animal experiments demonstrated that ovarian follicles exposed to high levels of FFAs had an abnormal ovarian steroidogenesis, reduced ovulation rates, and decreased oocyte competence for fertilization [9, 10]. However, the mechanism underlying FFA-induced cytotoxicity in GCs even resulting in oocyte maturation failure remains not fully understood.

Recently, some results demonstrated that the Akt/GSK3β signal pathway modulates the down-regulation of follicle stimulating hormone receptor (FSHR) expression in in the GCs of overweight/obese women [11], but the mechanism controlling this pathway is still unclear. Tribbles pseudokinase 3 (TRIB3) is the member of mammalian tribbles homologs, which coordinates crucial cellular processes, including lipid metabolism, apoptosis and cell stress, through interacting with different kinds of proteins. Compared with other two members of tribble family, TRIB3 protein levels were more precisely regulated in both a context- and microenvironment-dependent manner. Recent years, TRIB3 was actively investigated as a biomarker and therapeutic target for metabolic disease and cancer. Some studies have shown that TRIB3 can bind to a number of kinase-dependent proteins and dysregulate their function by negatively regulating their phosphorylation, thus affecting the activation of multiple signal transduction pathways, such as PI3K/AKT [12–15] and Notch [16]. Yan reported that palmitic acid (PA), an important FFA constituent in GCs can induce endoplasmic reticulum stress (ERs) in a human hepatic cell line, accompanied by a significant induction of tribbles of TRIB3 expression, which was associated with decreased levels of phosphorylated Akt (p-Akt) [12]. Moreover, some other reports showed that TRIB3 induces abnormal Akt phosphorylation under high PA conditions in various cells [13–15]. On the other hand, TRIB3 was involved in cell proliferation and fatty acid oxidation signaling in bovine cumulus cells, which are also derived from follicular cells as granulosa cells and played a key role in oocyte meiotic resumption regulation [17]. Therefore, we presumed that TRIB3 may participate in regulating FSHR expression in GCs. In this study, human GCs were obtained from follicular fluids in clinical infertile subjects and the TRIB3 effects were investigated on FSHR expression in GCs and relevant signal pathways. Meanwhile, some possible targets which can reduce the impact of high FFA levels on GCs function were also evaluated.

## Methods

### Subject enrollment

A total of 150 women undergoing IVF treatment due to tubal pathology were recruited from the Center for Reproductive Medicine, Ruijin Hospital, Shanghai Jiao Tong University School of Medicine. Patients who were more than 35 years of age or diagnosed with polycystic ovarian syndrome (PCOS, according to Rotterdam criteria [18]), endometriosis and other medical abnormality that could affect folliculogenesis were excluded from this research project. All participants gave their written informed consent regarding the use of clinical data, blood, and follicular fluid samples, and this study was approved by the ethics committee of Ruijin Hospital (2020104a). All patients underwent the standard GnRH antagonist protocol treatment with recombinant human FSH (rFSH; follitropin alfa; Merck, Geneva, Switzerland), which was started on the 2^nd^ day of the menstrual cycle.

### Collection of human granulosa cells and follicular fluid

Human follicular fluid was collected during oocyte retrieval. Only follicular fluid from follicles with diameters of 16–20 mm and free of blood upon macroscopic analysis were collected for further analyses. Each sample was centrifuged (250× *g*, 10 min,) and the supernatant was collected and stored at −80°C until analyzed. The pellets were resuspended with phosphate-buffered saline (PBS), and the suspension was layered over 40 % Percoll (Sigma-Aldrich; Merck KGaA) and centrifuged at 450 × *g*, 4°C for 20 min. GCs were collected from the interphase between follicular fluid and the percoll layer and washed with PBS thrice, then incubated with trypsin (ThermoFisher Scientific, Waltham, MA, USA) at 37°C for 2 min. Next, cells were incubated with red blood cell lysis buffer for 5 min at 4°C to lyse the surplus red blood cells. Finally, the GCs were cultured overnight in DMEM/F12 (Hyclone, Logan, UT, USA) medium supplemented with 10% fetal bovine serum (FBS, Gibco, Waltham, MA, USA) and 1% penicillin-streptomycin (Hyclone).

### Cell culture

GCs and human granulosa-like tumor (KGN) cell line (Feiya Biotechnology Co., Ltd. Jiangsu, China) were used for in vitro study. KGN cells were undifferentiated and maintained the physiological characteristics of human ovarian granulosa cells. Due to human primary GCs was hard to passage after transfection, we make KGN cells transfected with TRIB3-shRNA and negative control plasmids to investigate the mechanisms. All cell lines were cultured in DMEM/F12 supplemented with 10% FBS and 1% penicillin-streptomycin, and incubated at 37°C with 5% CO_2_.

### Lentiviral knockdown

TRIB3-shRNA and negative control (NC) plasmids were constructed by GeneChem Biotechnology Co., *Ltd* (Shanghai, China). KGN cells were transfected with the indicated lentiviruses (multiplicity of infection = 50) for 24 h. Then the transfected cells were selected using 1 μg/mL puromycin (Beyotime, Jiangsu, China) to generate stable cell lines. Western blotting analysis confirmed *TRIB3 k*nockdown in target cells.

### Cell treatments

In this study, palmitic acid (PA) (Sigma-Aldrich, St. Louis, MO, USA) was employed to investigate the FFAs effect on the GCs and KGN cells. PA was diluted with 5% FA-free BSA(Sigma) at 70°C (50 mM stock solutions) and p-Akt (Ser473) inhibitor Palomid 529 (P529) (Selleck, Houston, TX, USA) was dissolved with DMSO (100 mM stock solutions). Before each experiment, 50 mM PA was diluted in cell culture medium and used at a final concentration of 200 μM, and P529 was used at a final concentration of 60 μM in cell culture medium. Recombinant FSH (rFSH) was added to the culture dishes at a final concentration of 10 IU/mL to test the FSH-stimulated protein expression of genes in the FSHR signal pathway. To test the level of E2, 100 nM testosterone (Sigma) was added to the culture medium of shRNA-NC cells and shRNA-TRIB3 cells as a substrate for the synthesis of estrogen in vitro. Subsequently, collected human GCs with low FFAs level (FFA≤0.41mM/L) were divided into four groups and treated with the reagents as follows for 24 h, respectively: (a) Blank control (no intervention); (b) rFSH; (c) 200 μM PA + rFSH; (d) 300 μM PA + rFSH. shRNA-TRIB3 cells were divided into six groups and stimulated with reagents as follows for 24 h: (a) Blank control (no intervention); (b) rFSH; (c) 200 μM PA + rFSH; (d) 300 μM PA + rFSH; (e) 200 μM PA + rFSH + P529; (f) 300 μM PA + rFSH + P529. Meanwhile, KGN cells transfected with shRNA- NC were designated as the control.

### Laboratory analysis

Serum and follicular FFA concentrations were quantified as described previously [19]. Serum anti-Mullerian hormone (AMH) levels were measured with the Human AMH ELISA kit (Biotra, Guangzhou, China), and the levels of FSH, LH, E2, progesterone (P), and total testosterone were measured using a chemiluminescence immunoassay (ECLIA) kit (Beckman Coulter, Brea, CA, USA) according to the manufacturer’s instructions, respectively.

### RNA extraction and qRT-PCR

Real-time PCR assay was used to determine the mRNA expression levels of *ATF4, TRIB3, CYP19A1, CYP17A1*, and *FSHR*. Total RNA was isolated using a Takara RNA Extraction Kit (Takara, Dalian, China). cDNA was synthesized by reverse transcription using an RT reaction kit (Takara), according to the manufacturer's instructions. The SYBR Green qPCR Mix (Takara) was used to perform real-time quantitative PCR (qPCR). The cycling conditions included 30 min incubation at 95 °C, followed by 40 cycles at 95 °C for 5 s, 60 °C for 34 s and 95°C for 15 s (Applied Biosystems 7500, Fisher Scientific, USA). Meanwhile, *GAPDH* was used as an internal control. The 2^−ΔΔCt^ method was used to calculate relative expression levels (defined as fold-change). Each cell sample in every group was measured thrice and a *P*-value <0.05 was considered statistically significant. All primer sequences were list on Table 1. GAPDH was selected as a proper housekeeping gene, according to the NormFinder software (MOMA, Aarthus, Denmark). Results of NormFinder were listed on Table 2.

**Table 1:** Oligonucleotide primers used for qPCR analysis

Target gene	Primer sequence (5’ → 3’)	Sense Anti-sense*	Product size (bp)	Efficiency (%)
**FSHR**^a^	*GGC CAT GCT CAT CTT CAC TG*	S	157	
	*ATA GAG GAA GGG GTT GGC AC*	AS	157	98.84
***TRIB3***	GTCCGAGTGAAAAAGGCGTA	AS	158	
	TGCCCTACAGGCACTGAGTA	S	158	98.82
***ATF4***^b^	ATGACCGAAATGAGCTTCCTG	S	153	
	GCTGGAGAACCCATGAGG	AS	153	99.33
***CYP17A1***^a^	*GCT GCT TAC CCT AGC TTA TTT GT*	S	174	
	*ACC GAA TAG ATG GGG CCA TAT TT*	AS	174	98.03
***CYP19A1***^a^	*CGA AAG TGC TAT CGT GGT T*	S	178	
	*TGT GGA AAT CCT GCG TCT*	AS	178	98.84
***GAPDH***^a^	*CAC ATC GCT CAG ACA CCA TG*	S	198	
	*TGA CGG TGC CAT GGA ATT TG*	AS	198	98.84

Note: * S, sense; AS, anti-sense.

^a^XU P, HUANG B-Y, ZHAN J-H et al. Insulin Reduces Reaction of Follicular Granulosa Cells to FSH Stimulation in Women With Obesity-Related Infertility During IVF. The Journal of Clinical Endocrinology & Metabolism 2018, 104: 2547-2560

^b^DI F, LIU J, LI S et al. Activating transcriptional factor 4 correlated with obesity and insulin resistance in polycystic ovary syndrome. Gynecol Endocrinol 2019, 35: 351-355

**Table 2:** Result of NormFinder

Gene name	Group Difference	Group SD	Stability Value
**GAPDH**	0.06	0.12	0.1
**β-Actin**	0.35	0.19	0.26
**18s RNA**	0.3	0.26	0.27

### Immunofluorescence staining

Immunofluorescence staining was performed according to a previously described protocol [20]. Briefly, collected GCs were incubated for 24 h and then fixed in 4% paraformaldehyde for 30 min. After fixation, the cells were blocked with 3% bovine serum albumin (BSA, Servicebio, Wuhan, China) for 30 min. Then, the cells were incubated with rabbit anti-TRIB3 (cat. 3868, 1:200; Proteintech, Rosemont, IL, USA), rabbit anti-FSHR (cat. 22665-1-AP 1:200; Proteintech), rabbit anti-ATF4 (cat. GB111137, 1:200; Servicebio) antibody at 4°C overnight, followed by incubation with secondary antibodies AlexaFluro488 (cat. GB25303, 1:200; Servicebio) for 1 h in the dark. Finally, coverslips were mounted on slides with antifade mounting medium containing DAPI (Servicebio) after the slides were washed with PBS thrice. Three independent experiments were performed for each condition. Images were captured using a pannoramic desk (P250, 3D Histech, Hungary).

### Western blotting

Total protein from the GCs and KGN cells was obtained with radio-immunoprecipitation assay (RIPA, Beyotime, Jiangsu, China) lysis buffer containing 1% protease inhibitor cocktail (Roche, Basel, Switzerland), and protein concentrations were measured using the BCA protein assay kit (Beyotime) [21]. Then, 30 μg protein samples were loaded per well in 4%-12% Bis-Tris polyacrylamide gels (Tanon, 180-8008H) and separated by electrophoresis for 1 h at 120 V. Proteins were transferred onto polyvinylidene difluoride (PVDF) membranes (Millipore, Billerica, MA, USA) with transmembrane equipment (Tanon) for 50 min at 400 mA, 4^0^C. PVDF membranes containing proteins were blocked with protein free rapid blocking buffer (Epizyme, Cambridge, MA, USA) for 20 min and then incubated with specific primary antibodies overnight at 4°C. Primary antibodies included TRIB3 (1:5000, Abcam, Cambridge, UK), FSHR (1:1000, Proteintech), p-GSK3β (1:3000, Abcam), GSK3β (1:5000, Abcam), phospho-Akt (1:2000, CST), Akt (1:1000, Cell Signaling Technology [CST] Danvers, MA, USA), and tubulin *(*1:5000, Abcam). After washing with tris-buffered saline containing 0.1% of Tween-20 (TBST) for 10 min thrice, the PVDF membranes were incubated with horseradish peroxidase-conjugated anti-rabbit secondary antibody (1:5000, CST) for 1 h and target proteins were detected using the Western Chemiluminescent HRP Substrate Kit (Millipore), according to the manufacturer’s instructions. The results of Western blots were quantified by densitometry using ImageJ software (National Institutes of Health, Bethesda, MD, USA) and data were normalized as compared to the control treatment.

### Statistical analyses

The qPCR results were performed with 37 independent biological replicates, and for each biological replicate, three technical replicates were performed. Immunocytochemistry and western blot experiments consisted of at least 3 biological replicates, each containing 3 technical replicates. Quantified data are expressed as mean ± standard error of the mean (SEM). All statistical analysis was performed with SPSS version 24.0 (SPSS Inc., Chicago, IL, USA). After inspection for normal distribution of the data, student’s *t*-test was employed to compare two groups. Non-parametric Kruskall-Wallis (KW) test was employed to analyze differences between more than two groups. *P* < 0.05 was considered statistically significant.

## Results

### Basic characteristics of infertile subjects and IVF cycle outcomes

This study totally enrolled 150 women aged 24–35 years who were infertile due to tubal pathology. All the subjects were divided into two groups (highest quartile vs. other quartiles) based on their FFA levels in the follicular fluid samples: Group I, patients below the third quartile (FFA ≤ 0.41 mM/L, n =113); Group II, patients above the third quartile (FFA > 0.41 mM/L, n = 37) according to the method of group division [22]. As shown in Table 3, we found that the average FSH dose consumption per oocyte (FSH dose/oocyte) in Group II was higher than that in Group I. Moreover, the number of top-quality embryos in Group II was much less than that in Group I. However, there was no statistical significance when other parameters such as age, BMI, or ovarian reserve, were compared between the two groups.

**Table 3:** Comparison of basic characteristics of study subjects

Parameter	Group I (n = 113)	Group II (n =37)	P Value
Age (y)	30.6 ± 2.3	30.9 ± 2.1	0.77
BMI (kg/m2)	23.06 ± 3.75	23.09 ± 3.06	0.15
AMH (ng/mL)	2.9 ± 0.8	3.1 ± 1.0	0.41
Serum FFA (mmol/L)	0.38 ± 0.09	0.41 ± 0.11	0.29
Basic sex hormones			
FSH (IU/L)	8.9 ± 1.7	8.7 ± 2.0	0.38
LH (IU/L)	3.8 ± 2.1	4.0 ± 1.8	0.55
P (ng/mL)	0.71 ± 0.52	0.86 ± 0.41	0.89
E2 (pg/mL)	42.4 ± 11.7	45.9 ± 14.6	0.49
Total testosterone (ng/mL)	0.32 ± 0.11	0.37 ± 0.21	0.32
Ovarian stimulation cycle characteristics			
Total FSH dose (IU)	2325.5 ± 216.7	2623.0 ± 253.6	0.09
Duration of stimulation (d)	9.6 ± 0.9	10.1 ± 1.2	0.15
E2 on trigger day (pg/mL)	3418.3 ± 788.4	3077.5 ± 696.7	0.22
No. of oocytes retrieved	11.6 ± 2.1	10.8 ± 2.6	0.19
FSH dose (IU)/oocyte	200.4±22.6	242.8±25.4	0.04
Normal fertilization (%)	77.5 ± 8.4	72.3 ± 10.6	0.27
Viable embryo (%)	67.9 ± 12.3	65.2 ± 10.9	0.13
Top quality embryo (%)	25.9 ± 8.8	17.3 ± 10.2	0.04

Values are shown as mean ± SD. FSH dose /oocyte was calculated as FSH dose (IU) per number of retrieved oocytes. Abbreviations: BMI, body mass index; AMH, anti-Mullerian hormone; FSH, follicle-stimulating hormone; P, progesterone; E2, estradiol; LH, luteinizing hormone; FFA, free fatty acid

### Differences in mRNA and protein expression in GCs

**Fig. 1:**
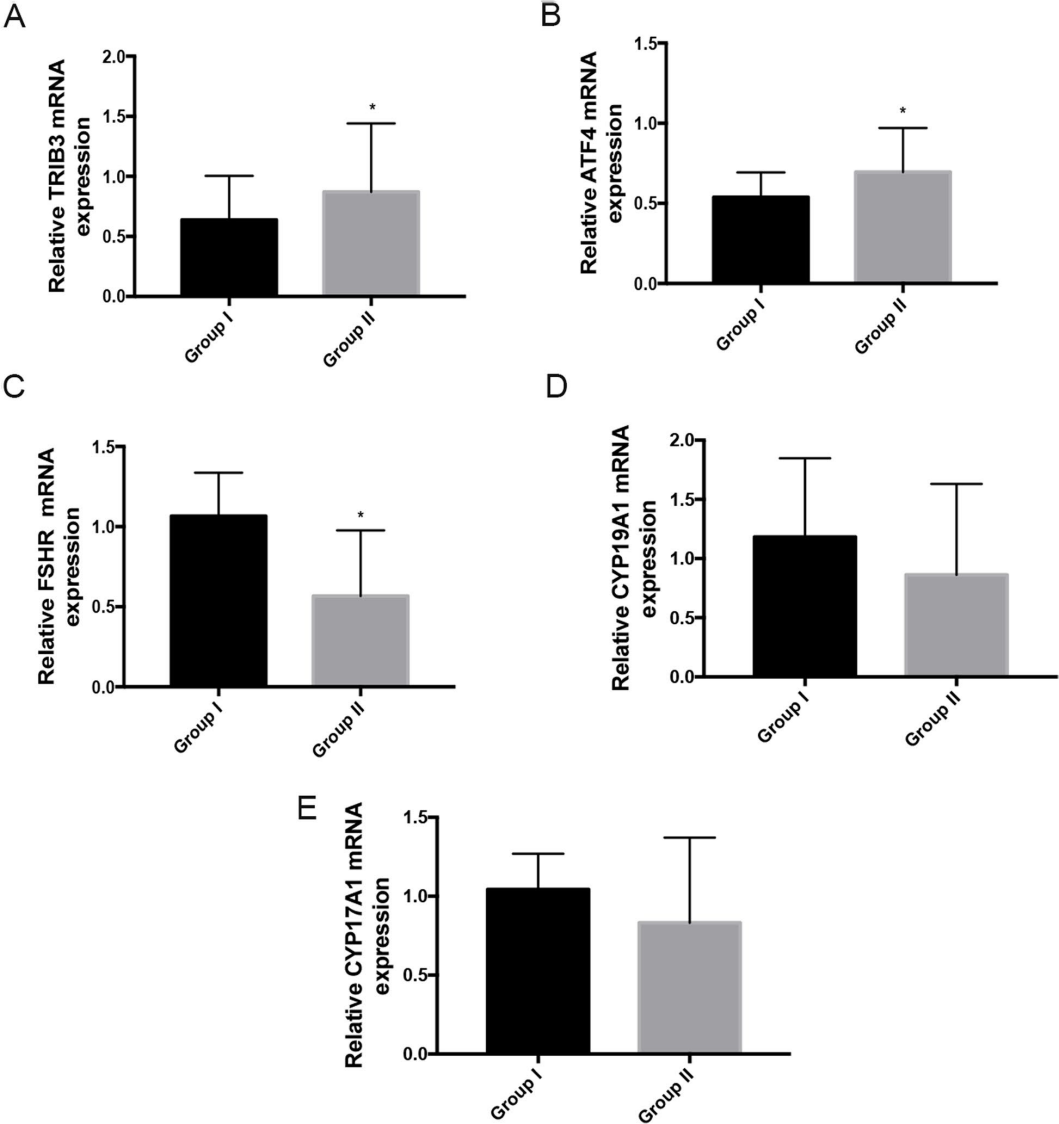
mRNA expression in GCs from 150 infertility women **A** Expression of TRIB3 was increased in patients of Group II. **B** Expression level of ATF4 was increased in patients of Group II. **C** Expression of FSHR was decreased in patients of Group II. **D**,**E** No obvious difference in mRNA expression levels of CYP19A1 and CYP17A1 were observed. Data are presented as mean ± SEM of three independent experiments. *P < 0.05 compared with of Group I

**Fig. 2:**
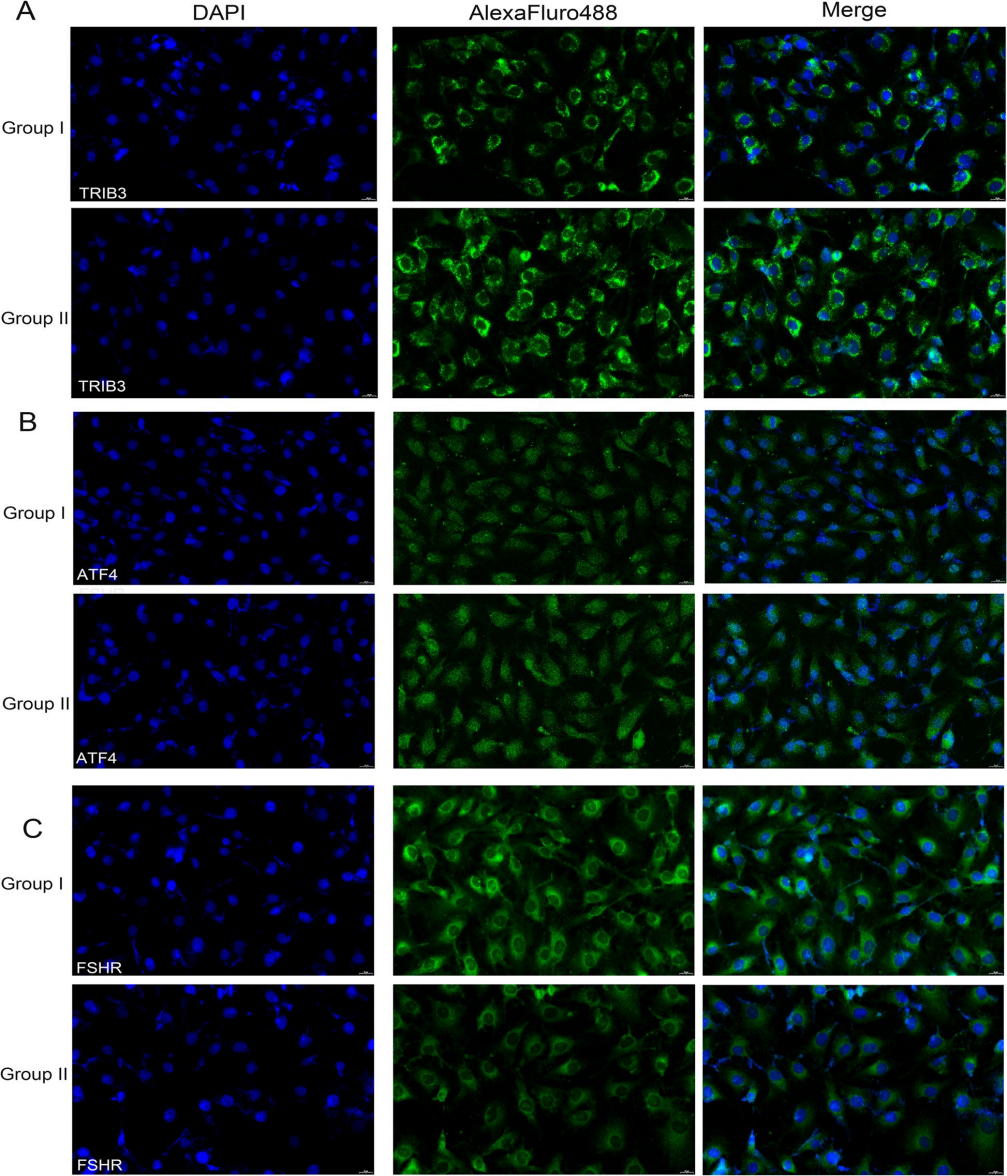
Protein expression of TRIB3 ATF4 and FSHR in GCs was detected by immunofluorescence. **A** Expression of TRIB3 protein was upregulated in patients of Group II. **B** Expression of ATF4 protein was upregulated in patients of Group II. **C** Expression of FSHR protein was downregulated in patients of Group II. AlexaFluro488 (green) was detected at the cell membrane in GC and cell nuclei were counterstained with DAPI (blue). All the images were acquired at 630× magnification. Scale bar: 20 μm

We measured the expression levels of related genes in all of the 150 GC samples. Compared with Group I, *TRIB3* and *ATF4* (Fig. 1A,B) expression levels were upregulated whereas the expression level of *FSHR* (Fig. 1C) was downregulated in Group II, but there were no differences in the expression of the steroid synthesis-related genes *CYP19A1* and *CYP17A1* between the two groups (Fig. 1D,E). Further, we used immunofluorescence analysis to detect the proteins of TRIB3, ATF4 and FSHR in human GCs. As shown in Fig. 2A, B, the protein levels of TRIB3 and ATF4 in Group II were also upregulated compared with that in Group I, while the expression of FSHR was downregulated (Fig. 2C).

### PA effects on TRIB3 and FSHR expression in vitro

**Fig. 3:**
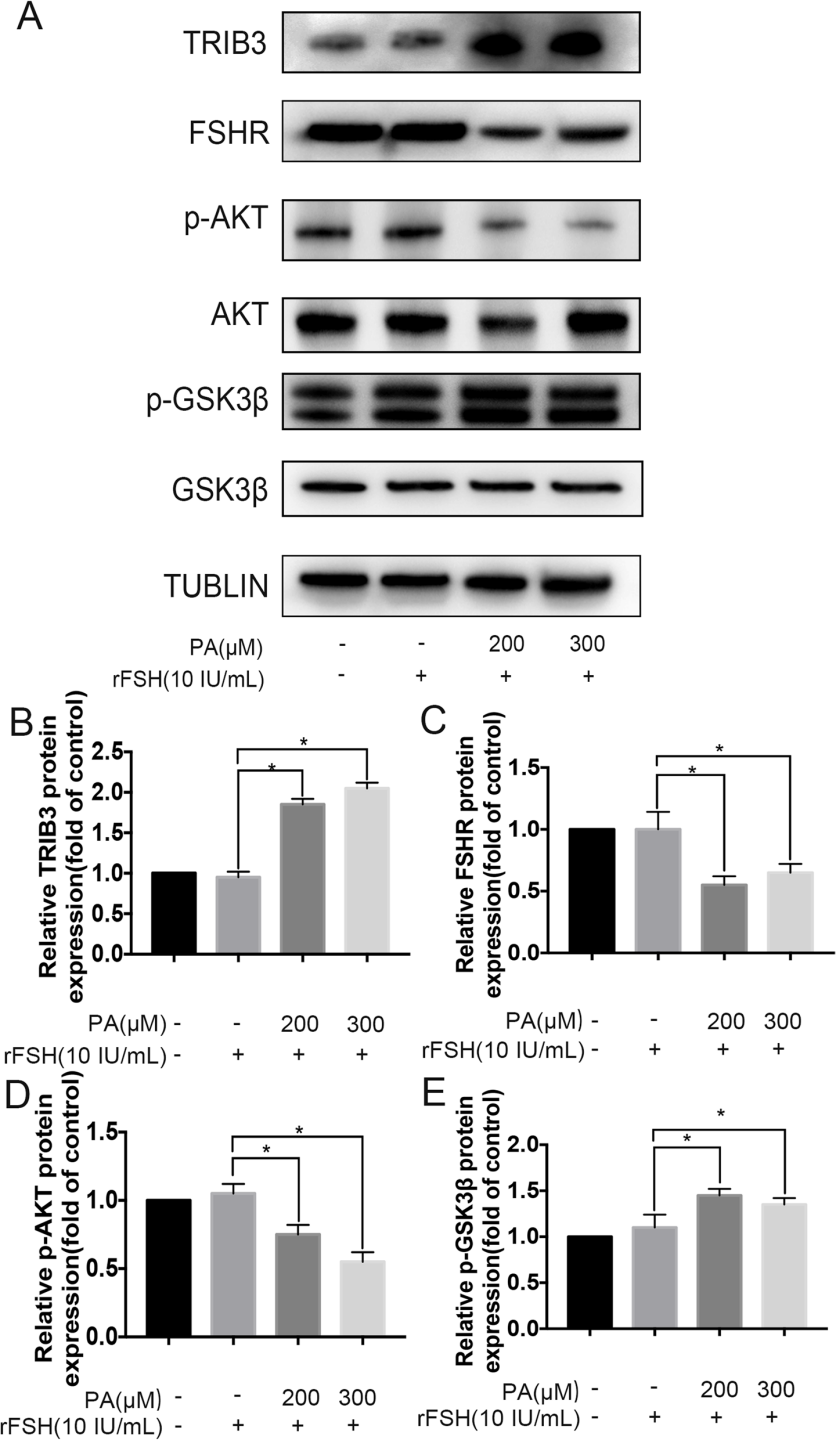
hGCs treated with PA in vitro. **A** Western blot analysis with tubulin used as the loading control. **B** PA increased TRIB3 levels in a concentration-dependent manner. **C** PA decreased FSHR levels in a concentration-dependent manner. **D**,**E** Reduced p-Akt and increased p-GSK3β protein levels indicating the Akt/GSK3β signaling was aberrantly activated in hGCs treated with PA. *P < 0.05

Based on previous work [23], we coincubated 200 μM PA, 300 μM PA and 10 IU/mL rFSH with the human GCs (FFA≤0.41mM/L) and then measured their protein expression levels, respectively (Fig. 3A). Western blotting analyses showed that TRIB3 protein expression level significantly increased in the PA-treated group (Fig. 3B). In contrast, the FSHR levels exhibited an opposite trend, which means that exposure to PA had concentration dependent inhibitory effects on FSHR expression levels (Fig. 3C). As shown in Fig. 3D, E, the expression level of p-Akt significantly decreased while the expression levels of p-GSK3β increased in the PA-treated group, while the levels of total AKT and GSK3β protein remained unchanged.

### TRIB3 Knockdown reverses dysregulated Akt/GSK3β signaling and declines in E2 production

**Fig. 4:**
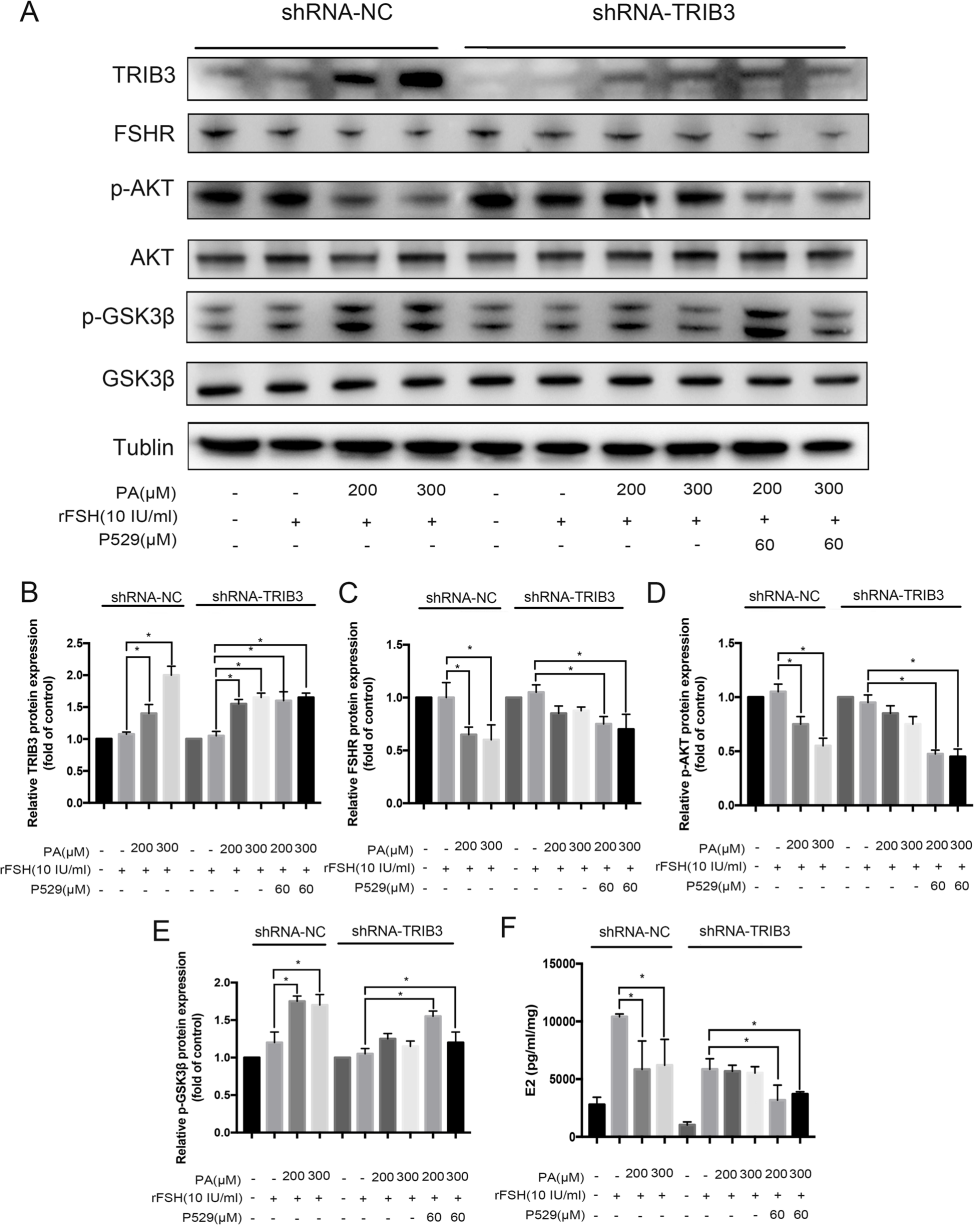
Western blot analysis of the Akt/GSK3β pathway following TRIB3 knockdown in KGN cells. **A** Western blot analysis was performed in shRNA-control and shRNA-TRIB3 cells treated with PA; tubulin was used as the loading control. **B** PA increased TRIB3 levels in a concentration-dependent manner, TRIB3 knockdown reduced this effect. **C** TRIB3 knockdown prevented PA-induced FSHR decrease. **D** TRIB3 knockdown blocked PA-induced decrease of p-AKT. **E** TRIB3 knockdown blocked PA-induced increase in p-GSK3β. (b–e) P529, together with rFSH and PA, enhanced the reduction of FSHR, p-Akt and the increase of p-GSK3β protein expression. **F** PA treatment inhibited E2 production, TRIB3 knockdown reversed this effect; P529 enhanced it

In human GCs, the high-FFA levels induced FSHR dysregulation and changed Akt/GSK3β signaling. However, it was unclear whether TRIB3 interferes either with FSHR expression or this effect occurs via Akt/GSK3β signaling. To clarify the underlying mechanism, we knocked down *TRIB3* gene and treated shRNA-TRIB3 cells with P529, an inhibitor of p-Akt (Ser473) formation. As shown in Fig. 4B, both shRNA-NC cells and shRNA-TRIB3 cells displayed significantly increased TRIB3 protein expression when treated with either 200 μM or 300 μM PA. *TRIB3* gene knockdown reversed the decreased expression level of FSHR in the PA-treated group (Fig. 4C), This result suggested that in the presence of P529 this inhibitory effect could be prevented.

On the other hand, *TRIB3* gene knockdown instead attenuated the downregulation of p-Akt induced by PA, and co-treatment with inhibitor P529 significantly suppressed Akt phosphorylation without altering total Akt expression (Fig. 4D). Moreover, TRIB3 gene knockdown reversed the rises in the p-GSK3β expression levels and this decline was inhibited in the presence of P529 (Fig. 4E), but the total GSK3β expression level remained unchanged. Meanwhile, PA treatment decreased the E2 levels in shRNA-NC cell culture supernatants (Fig. 4F). In addition, knocking down TRIB3 expression attenuated the inhibitory effects induced by palmitate on E2 expression levels, whereas inhibition of p-AKT formation with P529 addition reversed the dampened inhibitory effect of PA on E2 expression levels. Thus, these results strongly indicate that *TRIB3* knockdown can prevent PA-induced declines in E2 expression levels, whereas P529 co-incubation reversed the blunting effect of PA on E2 expression levels.

## Discussion

During oocyte maturation, the granulosa cells play an absolutely indispensable role in fulfilling this procedure. In the present study, some infertile subjects had higher levels of FFA in their follicular fluid than that in the other subjects. There are a growing number of studies in which it was reported that FFAs have detrimental effects on ovarian function. For instance, data from bovine cumulus cells showed that elevated follicular palmitic acid and stearic acid were associated with both impaired oocyte maturation, and fertilization rates as well as poor-quality embryos [24]. In IVF treatment, elevated follicular FFA levels were linked with poor cumulus-oocyte complex (COC) morphology, and they adversely influence ovarian follicular function [25]. Moreover, our previous work also found that the oocyte developmental competence had an association with FFA concentrations in follicular fluid from women undergoing IVF [19]. However, the mechanism of the FFA influence on the developmental potential of oocytes is still not fully elucidated.

The major components of FFAs in the follicular fluid from infertility women are oleic acid (31%), palmitic acid (27%), linoleic acid (25%) and stearic acid (12%) [25]. Amongst these FFAs, palmitic acid was considered as a major factor of lipotoxicity associated with endoplasmic reticulum (ER) stress formation [26]. Studies from bovine models demonstrated that in vitro exposure to pathophysiological palmitic acid concentrations can result in compromised oocyte quality and reduced oocyte developmental competence [27]. On the other hand, mouse oocytes were exposed to lipid-rich follicular fluid from patients, which can induce the dramatic decreased rate of mature oocytes in metaphase II (MII). Additionally, the expression of ER stress markers ATF4, ATF6, and GRP78 significantly increased in cumulus-oocyte complexes (COCs) which matured in lipid-rich follicular fluid [9]. The mechanism is that palmitic acid may inhibit the viability of mouse ovarian GCs and KGN cells through inducing ER stress and stimulating the cAMP/PKA/CREB signaling pathway [10]. In the present study, we found that ATF4 expression, one of the ER stress markers, significantly increased in the GCs from some women who underwent IVF treatment and had high FFA levels in their follicular fluid. Furthermore, we found decreased FSHR expression in the GCs from these women with high follicular FFA content, which may account for why the dose consumed of FSH per oocyte during ovarian stimulation was elevated. Furthermore, after IVF treatment the harvested embryos had poor quality in these women.

More importantly, we also observed that high follicular FFA levels upregulated the TRIB3 content of GCs. TRIB3 is a member of the pseudokinase family and several findings within the last decade indicated that it can differentially regulate many important metabolic processes, including lipid metabolism, oxidative stress, and ER stress [28]. As a novel ER stress-inducible gene, TRIB3 is induced via the ATF4–CHOP pathway and it plays a key role in cell apoptosis during ER stress [29]. Besides, the regulation of TRIB3 has been proven to be effective in antitumor contexts [30, 31].

TRIB3 mediates differential regulation of the degree of Akt phosphorylation in different cells. In endometrial cancer cells, TRIB3 enhanced cell apoptosis and suppressed cell proliferation and migration ability through inhibition of Akt [14]. However, TRIB3 promoted oral squamous cell carcinoma cell proliferation by increasing Akt phosphorylation [32]. Additionally, TRIB3 hyperexpression in rat muscle cells can impair phosphorylation of Akt [33]. In the current report, we show for the first time that there is a relationship between TRIB3 expression and Akt activity in human granulosa cells. We found that in both human granulosa cells and KGN cell line, PA (one main element in FFAs) stimulation increased TRIB3 expression. Such upregulation accompanied declines in the expression levels of p-Akt and FSHR. Knockdown of TRIB3 diminished the impact of PA on FSHR expression by increasing the phosphorylation of Akt (Ser473) and reducing p-GSK3β (Tyr216). Meanwhile, decreased E2 synthesis after PA treatment was rescued by TRIB3 knockdown in KGN cell line. Therefore, these results strongly suggest that TRIB3 has a crucial role in mediating PA-induced FSHR downregulation via Akt/GSK-3β signaling. Such control is also linked with impairment of E2 synthesis. Based on these results, we presume that TRIB3 is a functional upstream regulator of FSHR in human CGs.

The PI3K/Akt pathway is associated with the initiation and progression of a variety of cancers, including breast, colorectal, ovarian and pancreatic cancers, and endometrial carcinoma [34, 35]. As one of the first identified substrates of Akt, GSK3β was initially identified as an inhibitor of cellular responses to insulin by deactivating glycogen synthase phosphorylation, this enzyme can also influence cell division, growth, and development as an endogenous inhibitor of canonical wingless related integration site (Wnt) signaling [36, 37]. In patient-derived lymphoma cell lines, activated AKT phosphorylates GSK3β Ser9 which results in the inactivation of GSK-3β, which elevated p-β-catenin (Ser-675) [38]. On the other hand, phosphorylation of Tyr216 counteracts the inhibitory effect of Ser9 phosphorylation on GSK3β. GSK3β activity is instead facilitated by GSK3β/β-catenin and FSHR downregulation [11] . In this study it was shown that KGN cells under hyper-insulin treatment displayed reduced p-Akt along with increased p-GSK3β(Tyr216) levels. As a consequence, activated GSK3β downregulated FSHR expression through decreasing the phosphorylation status of β-catenin at sites Ser552 and Ser675.

During follicle development, FSH induces proliferation and steroidogenesis in GCs through binding to its receptor (FSHR) localized on GCs membrane. Impaired FSHR expression usually results in decreased GC responses to FSH stimulation. In this study, we found that the ovarian response was reduced in patients with high follicular FFA levels, which indicated higher consumption of gonadotrophin to obtain an oocyte. In the shRNA-NC cell culture supernatant, E2 production declined after PA-stimulation and this effect was reversed by *TRIB3* knockdown. On the other hand, treatment with P529 of TRIB3-shRNA cells decreased the E2 to levels that were below those obtained with PA stimulation. Therefore, inhibition of TRIB3 expression may be a new target to improve ovarian response under high FFA conditions.

In the current study, our results showed that TRIB3/Akt/GSK3β represents an important point of convergence and crosstalk between the PA response and FSHR expression. However, this study still has some limitations including our use of KGN cells as a *TRIB3* gene knockdown model. Although the KGN cell line is more likely to be closer to granulosa cells in normal physiological conditions than other human cell lines [39], additional studies are warranted because this model does not completely replicate the physiological environment of the human body. Furthermore, the TRIB3 effect on the granulosa cells or even on the folliculogenesis in conditional *TRIB3* gene knockout mouse model also needs further study.

## Conclusions

In summary, the present study demonstrated that the elevated FFAs in follicular fluid were associated with increased TRIB3 expression levels and reduced FSHR expression in human GCs. Palmitic acid, one main constituent of free fatty acids, can induce decreased FSHR expression levels via the TRIB3/Akt/GSK3β pathway in both human GCs and KGN cell line. This research is the first study to elucidate TRIB3 biological roles in GCs exposed to a high level of FFAs. Moreover, it also provides insight into a potential target for improvement of poor reproductive performance in women with metabolic disorders and female infertility treatment in clinics as well.

## Supplementary Information

**Additional file 1. **Diagram illustrates how TRIB3 regulates FSHR expression in granulosa cells under high levels of free fatty acids. (A)In PA-treated GCs and KGN cells, increased TRIB3 suppresses AKT activation. The inhibition of Akt on GSK3*β* activity was reduced, which may affect the transcriptional activity of *β* -catenin, resulting in subsequent decreased FSHR expression. (B)TRIB3 knockdown reversed declines in FSHR expression, which also resulted in increased p-Akt levels and declines in the p-GSK3β level. (C) Treatment of *TRIB3*-knockdown cells with an inhibitor of p-Akt (Ser473) resulted in rises in the levels of p-GSK3β as well as decreases of FSHR expression. Inhibition of TRIB3 expression may be a new target to improve ovarian response under high FFA conditions. FFA: free fatty acids; *p*: phosphorylation; TCF: transcription factor.

**Additional file 2.** Results of TRIB3 primer sequences blasting. The results show that the primer of TRIB3 have good specificity.

## Data Availability

The datasets used and analyzed during the current study are available from the corresponding author on reasonable request.

## Abbreviations

BMI: Body mass index
TC: Total cholesterol
TG: Triglycerides
FFAs: Free fatty acids
GCs: Granulosa cells
IVF: In vitro fertilization
PCOS: Polycystic ovary syndrome
ER: Endoplasmic reticulum
Akt: protein kinase B
Wnt: Wingless-related integration site
qPCR: Quantitative PCR
rFSH: Recombinant human FSH
T: Testosterone
TBST: Tris-buffered saline–Tween 20
p: phosphorylated
PI3K: Phosphatidylinositol 3-kinase
KGN: Human granulosa-like tumor cells
GSK3: Glycogen synthase kinase 3
GAPDH: Glyceraldehyde 3-phosphate dehydrogenase
FSHR: FSH receptor
E2: Estradiol
CYP19A1: Cytochrome P450 19A1
CYP17A1: Cytochrome P450 17A1
ATF4: Activating transcription factor 4
BSA: Bovine Serum Albumin
COCs: Cumulus-oocyte complexes
TRIB3: Tribbles pseudokinase 3
NEFAs: Non-esterified fatty acids
PA: Palmitic acid
PBS: Phosphate-buffered saline
FBS: Fetal bovine serum
AMH: Anti-Mullerian hormone
MII: Metaphase II
rFSH: Recombinant FSH
P529: Palomid 529
ECLIA: Chemiluminescence immunoassay
