# Supplementary material for: TRIB3 regulates FSHR expression in human granulosa cells under high levels of free fatty acids
Source: Reprod Biol Endocrinol. 2021 Sep 9;19:139. doi: 10.1186/s12958-021-00823-z (PMC8428109; doi:10.1186/s12958-021-00823-z)
Supplement: Supplementary file 1 — Additional file 1. Diagram illustrates how TRIB3 regulates FSHR expression in granulosa cells under high levels of free fatty acids. (A)In PA-treated GCs and KGN cells, increased TRIB3 suppresses AKT activation. The inhibition of Akt on GSK3β activity was reduced, which may affect the transcriptional activity of β -catenin, resulting in subsequent decreased FSHR expression. (B)TRIB3 knockdown reversed declines in FSHR expression, which also resulted in increased p-Akt levels and declines in the p-GSK3β level. (C) Treatment of TRIB3-knockdown cells with an inhibitor of p-Akt (Ser473) resulted in rises in the levels of p-GSK3β as well as decreases of FSHR expression. Inhibition of TRIB3 expression may be a new target to improve ovarian response under high FFA conditions. FFA: free fatty acids; p: phosphorylation; TCF: transcription factor. [file 12958_2021_823_MOESM1_ESM.pptx]

## Slide 1
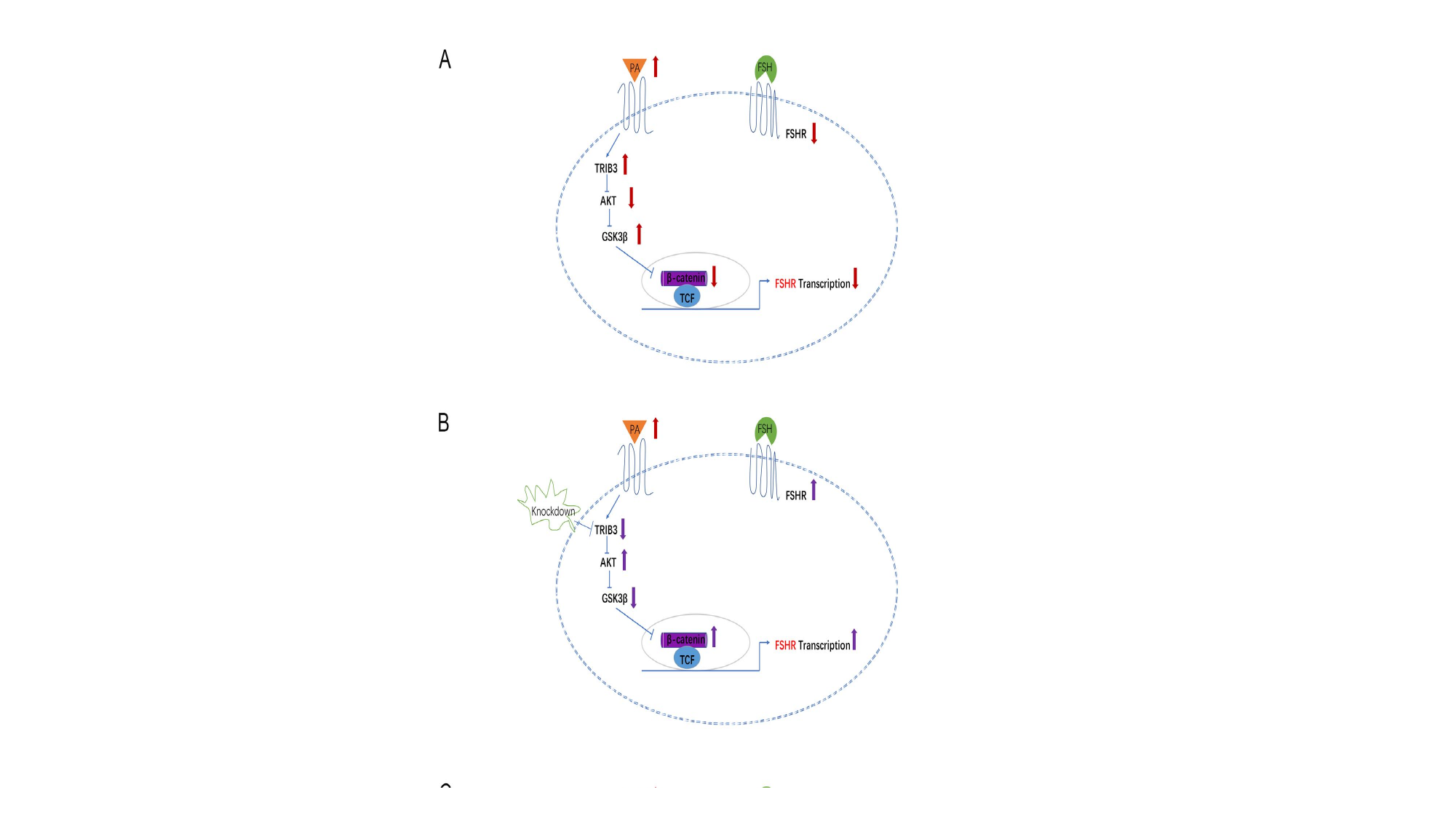

## Slide 2
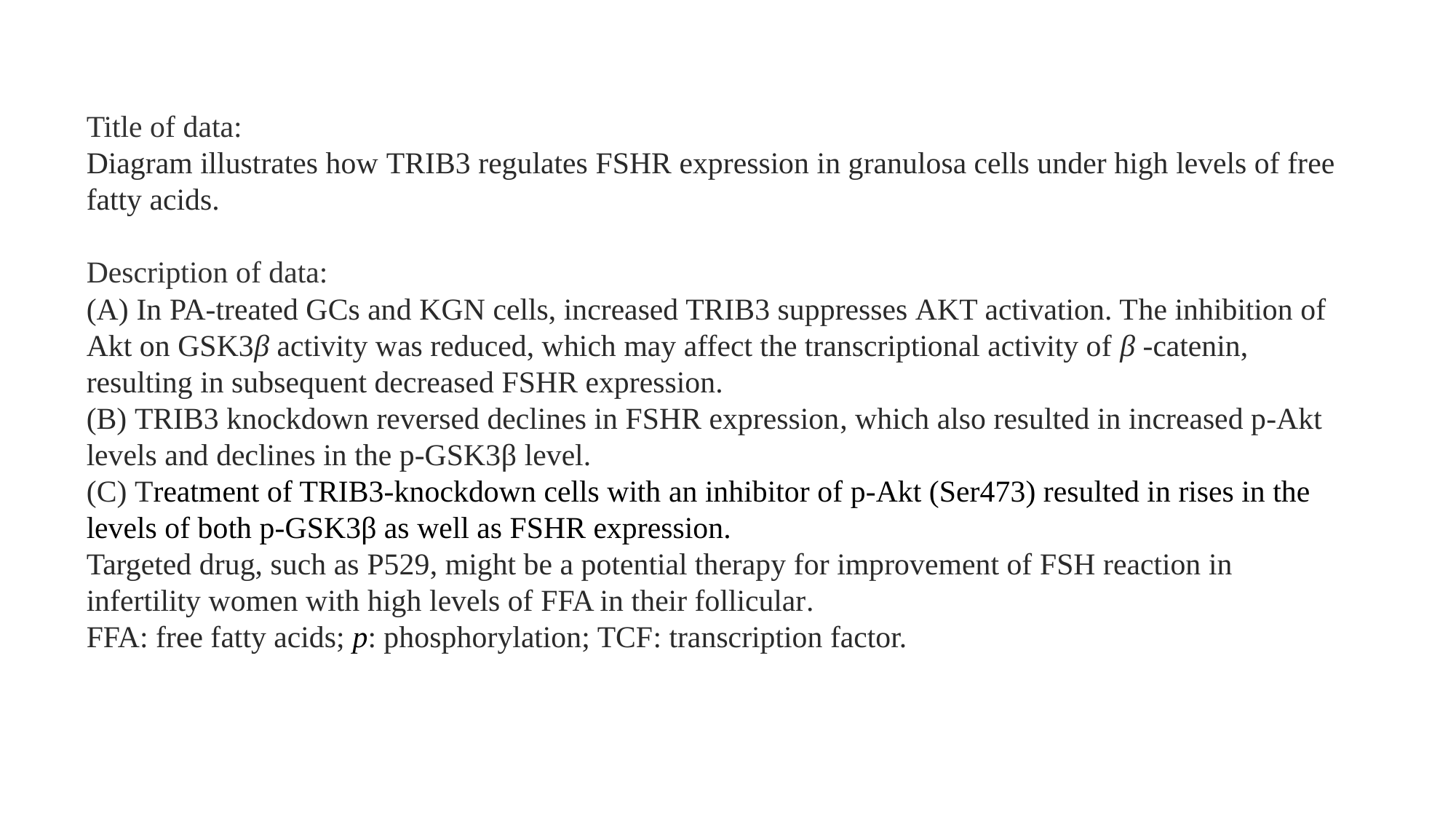

Title of data:
Diagram illustrates how TRIB3 regulates FSHR expression in granulosa cells under high levels of free fatty acids.
Description of data:
(A) In PA-treated GCs and KGN cells, increased TRIB3 suppresses AKT activation. The inhibition of Akt on GSK3β activity was reduced, which may affect the transcriptional activity of β -catenin, resulting in subsequent decreased FSHR expression.
(B) TRIB3 knockdown reversed declines in FSHR expression, which also resulted in increased p-Akt levels and declines in the p-GSK3β level.
(C) Treatment of TRIB3-knockdown cells with an inhibitor of p-Akt (Ser473) resulted in rises in the levels of both p-GSK3β as well as FSHR expression.
Targeted drug, such as P529, might be a potential therapy for improvement of FSH reaction in infertility women with high levels of FFA in their follicular.
FFA: free fatty acids; p: phosphorylation; TCF: transcription factor.
