# Supplementary material for: TRIB3 regulates FSHR expression in human granulosa cells under high levels of free fatty acids
Source: Reprod Biol Endocrinol. 2021 Sep 9;19:139. doi: 10.1186/s12958-021-00823-z (PMC8428109; doi:10.1186/s12958-021-00823-z)
Supplement: Supplementary file 2 — Additional file 2. Results of TRIB3 primer sequences blasting. The results show that the primer of TRIB3 have good specificity. [file 12958_2021_823_MOESM2_ESM.pptx]

## Slide 1
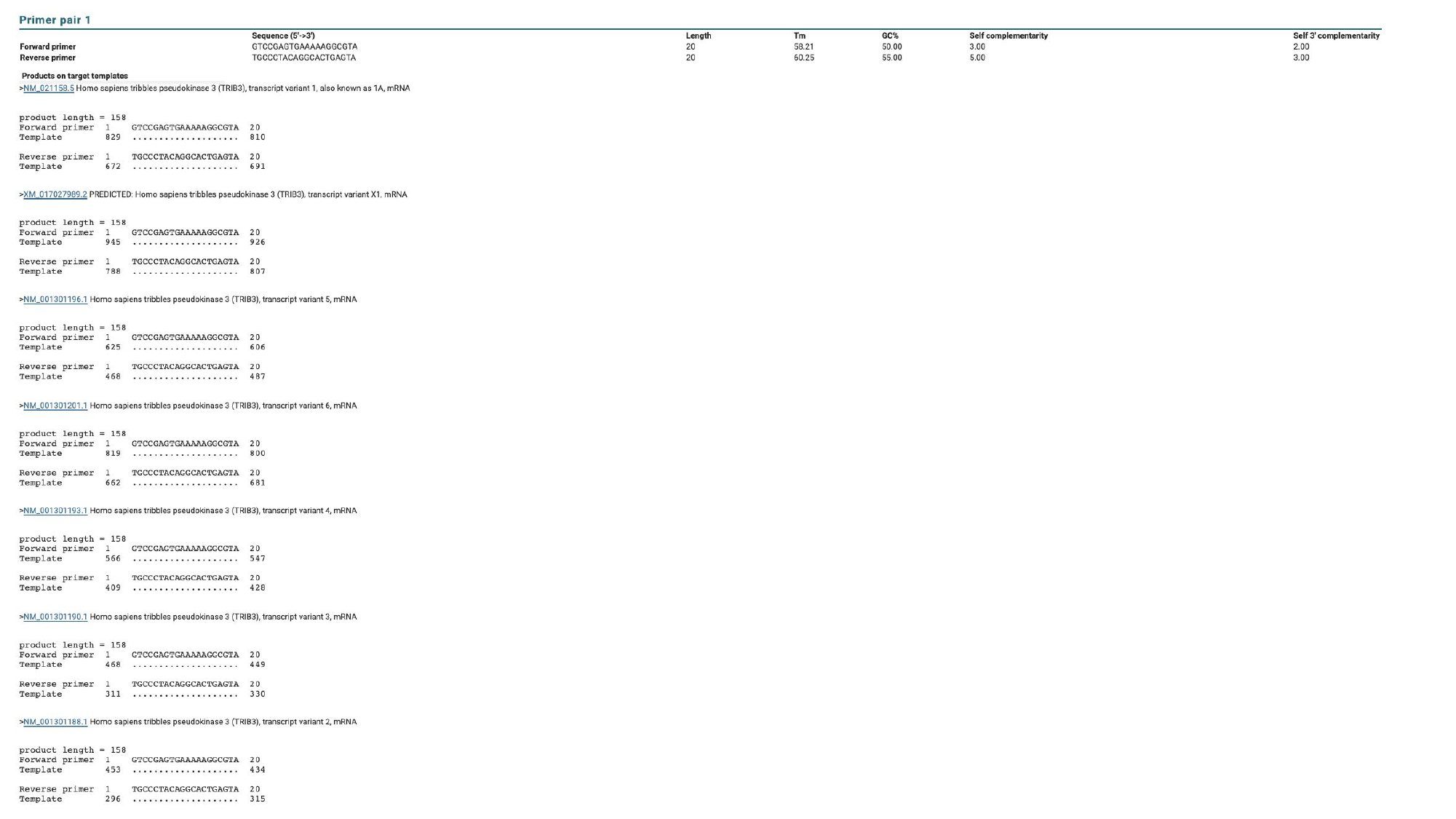

## Slide 2
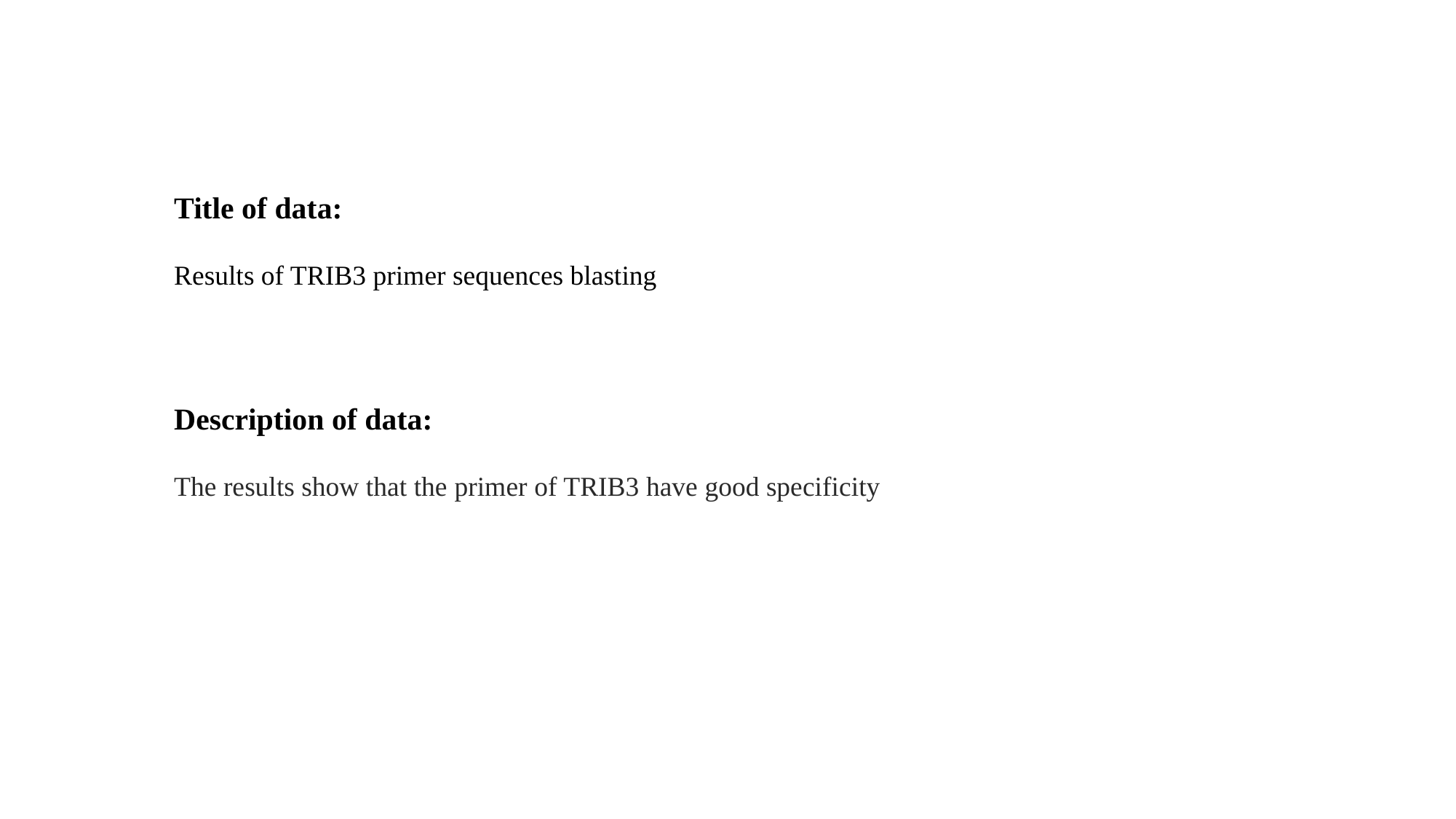

Title of data:
Results of TRIB3 primer sequences blasting
Description of data:
The results show that the primer of TRIB3 have good specificity
